# Spiritual needs and satisfaction with life: an exploration of mediating pathways

**DOI:** 10.1007/s00520-025-10105-8

**Published:** 2025-11-06

**Authors:** Gary Kwok, Alan B. Astrow, Daniel P. Sulmasy, Katie A. Devine

**Affiliations:** 1 Cancer Prevention Precision Control Institute, Center for Discovery & Innovation, Hackensack Meridian Health, 111 Ideation Way, Nutley, NJ 07110 USA; 2 Hematology and Medical Oncology, Department of Medicine, New York-Presbyterian Brooklyn Methodist Hospital, Weill Cornell Medicine, Brooklyn, NY 11215 USA; 3 The Kennedy Institute of Ethics, Departments of Philosophy and Medicine, Georgetown University, Washington, DC 20057 USA; 4 Pediatric Hematology/Oncology, Rutgers Cancer Institute of New Jersey, New Brunswick, NJ 08901 USA

**Keywords:** Supportive care, Patient satisfaction, Quality of life, Quality of health care, Spirituality

## Abstract

**Purpose:**

This study aims to examine the specific pathways through which spiritual needs are associated with satisfaction with life, focusing on the mediating roles of perceived quality of care and satisfaction with care among cancer patients.

**Methods:**

A cross-sectional survey was conducted with participants from four outpatient hematology/oncology clinics. Path analyses tested hypothesized relationships between spiritual needs and satisfaction with life, focusing on the mediating roles of perceived care quality and satisfaction with care.

**Results:**

A total of *N* = 727 participants were recruited, with a mean age of 59.0 years; the majority were female (*n* = 483; 67.8%). Path analysis supported a serial multiple mediation model: higher spiritual needs were indirectly associated with lower satisfaction with life via lower perceived quality of care and reduced satisfaction with care. Specifically, greater spiritual needs were linked to lower perceived quality of care (Fig. 1c: spiritual needs quality of care; *b* = -0.73, *p* < 0.001), which was associated with lower satisfaction with care (quality of care satisfaction with care; *b* = 0.26, *p* < 0.001) and in turn, with lower satisfaction with life (satisfaction with care satisfaction with life; *b* = 0.40, *p* < 0.001).

**Conclusions:**

Higher spiritual needs were indirectly associated with lower satisfaction with life through a sequential pathway involving reduced perceptions of quality of care and lower satisfaction with care. Addressing spiritual needs and concerns in clinical settings may, therefore, play a critical role in improving both care experiences and psychosocial outcomes for cancer patients.

## Background

Spiritual beliefs and concerns are essential to high-quality cancer care [1, 2] and quality of life [3] across all disease trajectories. Spirituality offers a lens through which patients make sense of illness, while spiritual needs reflect their pursuit of meaning and peace [4]. Distinguishing between the two is key for accurate measurement in health research. Defining spirituality only in favorable terms risks overlooking spiritual struggle and distress, which may impact health [5]. This is especially relevant in cancer care, where existential uncertainty can shape patients’ experiences beyond emotional well-being [6, 7].

Research has shown that unmet spiritual needs are prevalent among cancer patients regardless of religious affiliation and participation [8, 9]. For example, studies have shown that 90% of cancer patients have one or more spiritual needs [10, 11]. Many patients are interested in having their spiritual needs addressed by their physicians and/or other healthcare providers [12, 13]. Unmet spiritual needs are linked to lower quality of care [8, 9], patient satisfaction [8, 9, 14], and quality of life [9, 15, 16]. Improved spirituality can lead to higher satisfaction with life through lessening negative emotions (e.g., depression, anxiety, and anger) and increasing positive emotions such as hope, love, and happiness [17]. It is also important to note the differences between spirituality and religiosity. While religiosity is the search for significance through beliefs and practices within the context of institutions [18], spirituality extends beyond meaning, purpose, and transcendence [19], and any individual can experience spirituality [2] through vocation, family, or nature [19].

Since spiritual needs are integral to the cancer experience, healthcare providers play a critical role in supporting patients’ overall well-being. Addressing spiritual concerns and fostering spiritual well-being is essential to holistic, patient-centered care. This has been highlighted by established associations between spirituality, quality of care, and well-being found in prior research. For example, a systematic review finds spiritual well-being is positively associated with the patient’s quality of life [20]. Another study has found that increased spiritual needs are associated with less satisfaction with patient care and lower perceived quality of care among cancer patients [9]. Studies have also shown that higher quality of care and patient satisfaction are associated with better quality of life [21, 22]. The biopsychosocial-spiritual model offers a comprehensive framework for understanding the interconnected dimensions of patient care by integrating physical, psychological, social, and spiritual factors [23]. This holistic approach recognizes patients as beings-in-relationship, acknowledging that illness can disrupt not only biological functioning but also psychological, social, and spiritual well-being [23]. While this model has advanced our understanding of the components essential to delivering holistic care, the specific pathways linking these domains remain underexplored. In particular, the relationships among spiritual needs, perceived quality of care, satisfaction with care, and overall satisfaction with life have not been directly tested. These gaps underscore the need for further investigation into how these factors interact to influence patient outcomes. Further investigation into the directionality and mediating mechanisms among these factors may deepen our understanding of how holistic care can be optimized to support patient outcomes.

The present study investigated potential mediating factors linking spiritual needs to life satisfaction. Specifically, this study explored different potential exploratory pathways linking spiritual needs to satisfaction with life through the care patients receive. Overall, we hypothesized that greater spiritual needs would be related to lesser satisfaction with life, as supported by previous studies. Using a serial multiple mediation model, we hypothesized that this relationship would be mediated by the perception of quality of care and subsequent satisfaction with care.

## Methods

### Participants and procedure

The present study drew on data from a previously conducted investigation that explored the spiritual, psychosocial, and religious needs among a racially and ethnically diverse hematology/medical oncology patient population [9]. Participants were recruited from four outpatient sites in Brooklyn, New York: the Maimonides Cancer Center at Maimonides Medical Center (a public hospital; 53%), Coney Island Hospital (also a public hospital; 7%), and two private oncology practices—one located in a middle- to upper-middle-class neighborhood (21%), and the other in a diverse community with a large immigrant population (19%). Recruitment took place between November 2013 and November 2014. Recruitment details were reported in Astrow et al. [9]. Briefly, participants were eligible if they were 18 years and older, spoke English, Spanish, Russian, or Chinese, and presented for a routine visit (i.e., not initial evaluation). These four languages represent the most commonly spoken languages in the Brooklyn area and reflect the primary linguistic groups served by the participating clinical sites. Patients presenting for initial evaluations were excluded because they had not yet experienced enough of the care process to meaningfully assess their satisfaction with care or perceived quality of care.

A research assistant (RA) approached potential participants in the waiting room to assess their interest in the study. Interested individuals received a detailed explanation, and any questions were addressed before written informed consent was obtained. After obtaining written informed consent, participants completed a self-administered questionnaire in their preferred language. All survey items were translated using a standardized process, including translation and back-translation, followed by cognitive pre-testing to ensure clarity and cultural appropriateness [24].

### Ethical considerations

The Institutional Review Board at the main study site (Maimonides Cancer Center) approved this study (IRB# 12/04/XA06).

### Measures

#### Spiritual needs

Spiritual needs were assessed using the subscale from the Spiritual Needs Assessment for Patients (SNAP) scale [25]. The Spiritual Needs subscale contains 13 measures of spiritual needs. Sample items include “How much would you like help with finding meaning in your experience of illness?” or “How much would you like to talk with someone about the meaning and purpose of human life?” All items were rated on a 4-point Likert scale (1 = “not at all” to 4 = “very much”). The scale is scored by summing all 13 items, resulting in a total score range of 13 to 52, with higher scores indicating greater needs. Prior research has demonstrated strong internal consistency (Cronbach’s *α* = 0.95) and established the scale’s validity [25].

#### Satisfaction with life

Satisfaction with life was assessed using the 5-item Satisfaction with Life Scale [26]. Sample items include “In most ways my life is close to my ideal” and “The conditions of my life are excellent.” Participants rated the items on a 7-point Likert scale (1 = “strongly disagree” to 7 = “strongly agree”). Total scores range from 5 to 35, with higher scores indicating higher levels of satisfaction with life. Scores can be interpreted using the following categories: 31–35 = extremely satisfied, 26–30 = satisfied, 21–25 = slightly satisfied, 20 = neutral, 15–19 = slightly dissatisfied, 10–14 = dissatisfied, and 5–9 = extremely dissatisfied [27]. The Satisfaction with Life scale has demonstrated strong internal consistency in prior studies with cancer patients (Cronbach’s *α* = 0.91) and has been validated in this population [28].

#### Satisfaction with care and perception of quality of care

Satisfaction with care and perception of quality of care was measured using the Quality of End-of-Life Care and Satisfaction with Treatment (QUEST) scale [29]. The Satisfaction with Care subscale has 6 items. Sample items include “How satisfied have you been with your doctor’s bedside manner or common courtesy?” Participants rated the items on a 5-point Likert scale (1 = “very dissatisfied” to 5 = “very satisfied”). Total scores range from 6 to 30, with higher scores indicating greater satisfaction with care. Reported internal consistency is high, with Cronbach’s *α* ranging from 0.88 to 0.95 [29].

The Perception of Quality of Care subscale contains 9 items. Sample items include “How often have the doctors spent enough time with you or arrived late when they promise to come see you?” Participants rated the items on a 5-point Likert scale (1 = “never” to 5 = “always”). Five reversed items were recoded before computing the total score. Scores range from 9 to 45, with higher scores reflecting higher perceived quality of care. Cronbach’s *α* for this subscale ranges from 0.83 to 0.88. Both subscales have demonstrated good validity and reliability in prior research [29].

### Data analysis plan

Path analysis was conducted using STATA 14 [30]. We first tested the satisfaction with care as a mediator (model 1: spiritual needs ➔ satisfaction with care ➔ satisfaction with life). In model 2, we tested the perception of quality of care as a mediator (i.e., spiritual needs ➔ quality of care ➔ satisfaction with life). In model 3, we tested the full mediation through the perception of the quality of care and satisfaction with care (i.e., spiritual needs ➔ quality of care ➔ satisfaction with care ➔ satisfaction with life).

Model fit was evaluated using the root mean square error of approximation (RMSEA), standardized root mean residual (SRMR), comparative fit index (CFI), and Tucker–Lewis index (TLI). Acceptable model fit was indicated by RMSEA and SRMR values below 0.10 (moderate) or 0.06 (good) and CFI and TLI values above 0.90 (good) or 0.95 (excellent) [31–36]. We excluded *n* = 123 patients with five or more missing items (i.e., ≥ 40% missing) on the SNAP spiritual needs subscale from the analysis [9]. This threshold aligns with common practices in survey research, where exclusion is recommended when a substantial proportion of scale items are unanswered [37]. For those patients who failed to complete one to four items, we imputed their missing values using the Multivariate Imputation by Chained Equations (MICE) package from R [38]. Bootstrapping was used to obtain 95% confidence intervals to assess the significant indirect effects.^1^ The model tested included race, gender, and diagnosis as covariates. Race/ethnicity, gender, and cancer diagnosis were included as covariates based on prior evidence suggesting their influence on patient satisfaction and care experiences.

## Results

A total of 727 patients participated in the study (M = 59.0 years, SD = 16.8). The majority were female (67.8%) and approximately half identified as non-Hispanic White (49%), followed by Black (25%), Asian (14%), and Hispanic (13%). Most participants reported English as the primary language spoken at home (57%), with additional representation from Russian (15%), Chinese (11%), and Spanish (9%) speakers. Regarding education, 10.3% of participants had not completed high school, and 47% were married. The most commonly reported diagnoses were breast cancer (23%), lung cancer (8%), and colon cancer (7%). Other diagnoses included ovarian cancer (3.2%), prostate cancer (2.9%), blood cancers (9.5%), low blood count (15.7%), bleeding or clotting disorders (4.7%), and other conditions (26.3%). A full description of the sample is available in the parent study [9].

Descriptive statistics and bivariate correlations are presented in Table 1. Higher spiritual needs were associated with lower satisfaction with life (*r* = −0.11, *p* < 0.01), lower satisfaction with care (*r* = −0.13, *p* < 0.001), and worse perception of quality of care (*r* = −0.13, *p* < 0.001). Satisfaction with life was positively correlated with both satisfaction with care (*r* = 0.23, *p* < 0.001) and perceived quality of care (*r* = 0.19, *p* < 0.001). Additionally, satisfaction with care was strongly positively correlated with perceived quality of care (*r* = 0.48, *p* < 0.001).

**Table 1:** Correlations and descriptive statistics of study variables

	Variable	1	2	3	4	*M* (SD)	Range	α
1	Spiritual needs	–				30.18 (11.60)	13–52	0.94
2	Satisfaction with life	−0.11**	–			22.96 (7.27)	5–35	0.89
3	Satisfaction with care	−0.13***	0.23***	–		22.23 (3.16)	5–25	0.93
4	Perception of quality of care	−0.13***	0.19***	0.48***	–	37.98 (5.88)	13–45	0.76

^*^p < 0.05; **p < 0.01; ***p < 0.001

Table 2 shows the model fit statistics for each model: model 1, satisfaction with care as mediator (i.e., spiritual needs ➔ satisfaction with care ➔ satisfaction with life); model 2, perception of quality of care as mediator (i.e., spiritual needs ➔ quality of care ➔ satisfaction with life); and model 3, full mediation through the perception of quality of care and satisfaction with care (i.e., spiritual needs ➔ quality of care ➔ satisfaction with care ➔ satisfaction with life). Model fit indices are summarized as follows: All models demonstrated good to excellent fit based on RMSEA values (ranging from 0.04 to 0.07) and excellent fit according to SRMR values (all < 0.05). For the CFI, models 1 and 3 met the threshold for good fit (> 0.90), whereas model 2 showed poor fit (CFI = 0.68). None of the models achieved acceptable fit based on the TLI, with all values falling below 0.90.

**Table 2:** Model fit statistics

Model	*χ*^2^	df	*χ*^2^/df	RMSEA	90% CI		CFI	TLI	SRMR
1	10.88	6	1.81	0.04	0.00	0.07	0.90	0.85	0.03
2	22.78	6	3.80	0.07	0.04	0.09	0.68	0.52	0.04
3	25.93	9	2.88	0.05	0.03	0.08	0.93	0.89	0.04

Acceptable model fit was defined as RMSEA and SRMR values below 0.10 (moderate fit) or 0.06 (good fit), and CFI and TLI values above 0.90 (good fit) or 0.95 (excellent fit)

The model’s coefficients for all the paths are presented in Table 3. Both model 1 (spiritual needs → satisfaction with care → satisfaction with life; estimate = −0.016, *95% CI* = −0.029 to −0.003) and model 2 (spiritual needs → quality of care → satisfaction with life; estimate = −0.015, *95% CI* = −0.025 to −0.004; see Table 3) demonstrated similar significant specific indirect effects. In the full model (model 3), which tested the serial pathway from spiritual needs → quality of care → satisfaction with care → satisfaction with life, the total indirect effect was also significant (estimate = −0.020, *95% CI* = −0.034 to −0.006; see Table 3). However, the individual indirect pathways in model 3—through satisfaction with care alone (estimate = −0.005, *95% CI* = −0.014 to 0.003) and through quality of care alone (estimate = −0.007, *95% CI* = −0.016 to 0.002)—were not statistically significant.

**Table 3:** Path estimates and bias‐corrected 95% confidence intervals for all models

Path	Estimate	*p*	Bootstrap 95% CI	
**Model 1**				
Total effect				
Spiritual ➡ Sat w/Life	−0.068	0.006	−0.117	−0.019
Direct effect				
Spiritual ➡ Sat w/Life	−0.053	0.031	−0.100	−0.005
Specific indirect effect				
Spiritual ➡ Sat w/Care ➡ Sat w/Life	−0.016	0.015	−0.029	−0.003
**Model 2**				
Total effect				
Spiritual ➡ Sat w/Life	−0.069	0.006	−0.117	−0.020
Direct effect				
Spiritual ➡ Sat w/Life	−0.054	0.031	−0.103	−0.005
Specific indirect effect				
Spiritual ➡ QoC ➡ Sat w/Life	−0.015	0.005	−0.025	−0.004
**Model 3**				
Total effect				
Spiritual ➡ Sat w/Life	−0.068	0.006	−0.117	−0.020
Direct effect				
Spiritual ➡ Sat w/Life	−0.048	0.048	−0.096	0.000
Specific indirect effect				
Spiritual ➡ Sat w/Care ➡ Sat w/Life	−0.005	0.228	−0.014	0.003
Spiritual ➡ QoC ➡ Sat w/Life	−0.007	0.115	−0.016	0.002
Spiritual ➡ QoC ➡ Sat w/Care ➡ Sat w/Life	−0.008	0.013	−0.014	−0.002
Total indirect effect	−0.020	0.004	−0.034	−0.006

**Fig. 1:**
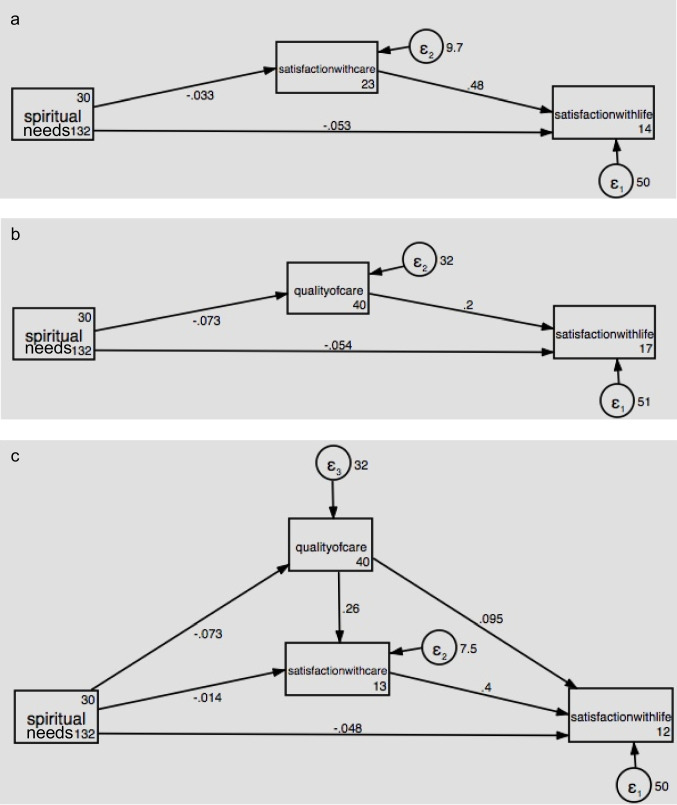
Hypothesized structural models and standardized path coefficients from the results of our analysis of the mediating effects of satisfaction with care and perception of quality of care on the relationship between spiritual needs and satisfaction with life. (**a**) Spiritual needs ➔ Satisfaction with care ➔ Satisfaction with life, (**b**) Spiritual needs ➔ Quality of care ➔ Satisfaction with life, (**c**) Spiritual needs ➔ Quality of care ➔ Satisfaction with care ➔ Satisfaction with life

The relationship between spiritual needs and satisfaction with life was significantly mediated by both perceived quality of care and satisfaction with care (spiritual needs ➔ quality of care ➔ satisfaction with care ➔ satisfaction with life; estimate = −0.008; *95% CI* = −0.014 to −0.002; see Table 3). As shown in Fig. 1c, greater spiritual needs were associated with a lower perception of quality of care (*b* = −0.73, *p* < 0.001), which, in turn, was associated with lower satisfaction with care (*b* = 0.26, *p* < 0.001; perception of quality of care satisfaction with care) and consequently associated with lower satisfaction with life (*b* = 0.40, *p* < 0.001; satisfaction with life satisfaction with life). In sum, the total effect of spiritual needs on satisfaction with life was significant (estimates = −0.068, *95% CI* = −0.117 to −0.020; see Table 3). We also tested an alternate model with satisfaction with care preceding perception of quality of care (i.e., spiritual needs ➔ satisfaction with care ➔ quality of care ➔ satisfaction with life; not shown in Fig. 1), and it was not significant (estimate = −0.003; *95% CI* = −0.006 to 0.001; not shown in Table 3).

## Discussion

### Summary of findings

This study examined the pathways through which spiritual needs, quality of care, and patient satisfaction with care influence patients’ satisfaction with life. We explored the relationships between spiritual needs and patient care and found support for mediation hypotheses that spiritual needs were indirectly associated with patients’ satisfaction with life through their perception of the quality of care they had received, which led to satisfaction with the care. Specifically, greater spiritual needs were associated with a lower perception of quality of care, which, in turn, was associated with lower satisfaction with care, resulting in lower satisfaction with life. The result aligned with the current literature suggesting that spiritual needs are negatively associated with satisfaction with care and perceived quality of care [9], while quality of care and patient satisfaction are positively associated with patient’s quality of life [21, 22].

### Interpretation

A cancer diagnosis can be a daunting experience, and the role of spirituality is closely tied to patients’ interpersonal relationships with providers and the psychosocial care available [39]. Patient-provider relationships’ spiritual and interpersonal significance has substantial implications on patients’ satisfaction and quality of life. We also found that satisfaction with care (model 1) and the perception of quality of care (model 2), when examined independently, were not associated with satisfaction with life. This suggests that these factors do not operate as isolated mediators. Instead, their influence on satisfaction with life is interconnected—patients who perceive their care to be of low quality are more likely to feel dissatisfied with their care experience, contributing to lower overall satisfaction with life. This sequential relationship highlights the importance of understanding how perceptions of care quality shape emotional responses to care, ultimately affecting broader well-being outcomes. To further explore the directionality of this relationship, we tested an alternative model in which satisfaction with care precedes quality of care (i.e., spiritual needs ➔ satisfaction with care ➔ quality of care ➔ satisfaction with life). This sequence did not yield significant findings, reinforcing the conceptual argument that patients’ perceptions of care quality likely inform, rather than follow, their overall satisfaction with care. This aligns with prior research suggesting that perceived quality often forms the cognitive foundation upon which emotional evaluations—such as satisfaction—are built [40, 41].

Unlike prior studies that examined spiritual needs and life satisfaction as independent or parallel constructs, our study identified a sequential pathway linking these variables through patients’ experiences of care. Importantly, perceptions of care quality reflect patients’ views of how their providers deliver care—including whether providers recognize and address their spiritual concerns. When providers attend to spiritual needs, patients may perceive their care as more holistic and responsive, enhancing their overall satisfaction with care. By situating spiritual needs within a care process framework, our findings underscore the importance of addressing spirituality not as an abstract construct but as a meaningful factor shaping patients’ real-time evaluations of care. This study extends existing research by clarifying a mechanism through which spiritual distress may diminish satisfaction with life, emphasizing the value of spiritually sensitive care in fostering positive health experiences and outcomes.

### Clinical implications

Our findings showed that increased spiritual needs have important associations with life satisfaction, specifically through the provision of care. Healthcare and psychosocial providers (e.g., oncologists, nurses, social workers, psychologists, etc.) working with racially and ethnically diverse cancer patients should pay attention to patients’ spiritual needs because it impacts how patients view and experience the care they receive. Patients with low levels of spiritual well-being often express hopelessness and may have more frequent follow-up visits [42]. Research has also shown that patients think it is appropriate for providers to inquire about their spiritual needs and other religious beliefs as part of their complete psychosocial history [8, 9]. In our study sample, patients’ responses suggest receptivity to clinical practices related to spiritual care, particularly when framed within a holistic care approach. Within this context, providers can play a meaningful role in improving patients’ quality of life by addressing their spiritual needs.

Clinical providers can play a critical role in supporting patients’ spiritual needs by encouraging open, culturally sensitive conversations as part of the psychosocial history. Tools such as the “FICA” framework (F: faith and beliefs; I: importance; C: community; A: address in care) can guide providers in initiating these discussions [43] alongside other open-ended questions to explore patients’ sources of meaning and spiritual concerns [44]. Active listening, presence, and empathetic inquiry are essential skills in this process [45].

Importantly, providers should recognize that addressing spirituality does not mean offering spiritual advice themselves. Instead, their role includes acknowledging its relevance to care and facilitating access to appropriate support. For example, referrals to chaplains—who are trained to assess and address issues like distress, death anxiety, and meaning-making—can be instrumental [46, 47]. Chaplains may also help patients reconnect with their spiritual community and collaborate with the care team by documenting key insights and recommendations in the medical record [48]. Streamlining communication and clarifying roles within the clinical team ensures that spiritual needs are addressed holistically without burdening providers or patients with unclear expectations. For psychologists and social workers, addressing spiritual needs is integral to understanding and supporting the whole person. When left unacknowledged, these needs can contribute to psychological and physical symptoms such as depression, anxiety, and pain [11, 49, 50]. Integrating questions about spirituality into clinical conversations allows psychosocial providers to gain deeper insight into the underlying meaning of patients’ concerns and experiences [51]. By drawing on their therapeutic skills and collaborating closely with chaplains and other care team members, psychosocial providers can help ensure that spiritual distress is recognized and addressed as part of comprehensive cancer care [52].

### Study limitations

The current study has a few limitations. Most notably, we could not control for cancer stage or prognosis, as these variables were not collected in the parent study. This omission is critical because patients with advanced-stage disease may experience heightened spiritual needs, potentially confounding the associations observed between spiritual needs, perceptions of care, and satisfaction with life. Spiritual concerns often intensify near the end of life, and without data on the disease stage or expected prognosis, we cannot determine whether the patterns we observe differ by severity or stage of illness. In addition, our assessment of spiritual needs, while based on validated instruments, may not fully capture culturally specific expressions of spirituality, which can vary significantly across diverse racial and ethnic groups. Spirituality is a deeply personal and culturally embedded construct; studies have shown that cultural background shapes how individuals experience, articulate, and cope with spiritual concerns. Thus, our findings may not reflect the full range or depth of spiritual needs in underrepresented or minority populations. This limitation also highlights the need for culturally sensitive measures and mixed-methods approaches better to understand the nuances of spiritual needs across diverse groups. Other limitations include the cross-sectional design, which precludes causal inferences, and the use of secondary data, which limited our ability to include other relevant covariates. For example, our path models did not account for participants’ specific religious affiliations, frequency of religious service attendance, or educational attainment—all of which may influence how spiritual needs are experienced and how care is evaluated.

### Future directions

Future research should explore strategies to overcome common barriers to assessing and addressing spiritual needs in clinical care, such as limited time, insufficient provider training in spiritual assessment, and variability in providers’ spiritual awareness or inclinations [52]. Implementation science approaches can play a critical role in identifying and addressing these multilevel barriers—from individual provider attitudes to organizational and systemic challenges. For example, studies could examine how best to integrate spiritual assessments into routine workflows, tailor training interventions for diverse clinical disciplines, and evaluate the fidelity and sustainability of spiritual care practices. One promising area of implementation research includes testing chaplain referral systems—such as standardized pathways for automatically referring patients with elevated spiritual needs to trained chaplains or spiritual care providers—as a means to operationalize spiritual care within healthcare delivery.

At the policy level, future work could focus on incorporating spiritual care into clinical guidelines and quality standards, advocating for institutional support, and expanding reimbursement mechanisms for providers addressing spiritual concerns. Developing scalable and culturally sensitive training programs for healthcare and psychosocial providers will also ensure that spiritual needs are consistently recognized and met across diverse patient populations.

## Conclusions

This study identified a significant indirect pathway through which higher spiritual needs were associated with lower satisfaction with life, mediated by lower perceived quality of care and reduced satisfaction with care. These findings underscore the potential impact of unmet spiritual needs on patient experiences and psychosocial well-being. Incorporating spiritual needs assessments and interventions into routine cancer care may improve patients’ perceived care quality and overall life satisfaction.

## Data Availability

The data that support the findings of this study are available from the corresponding author upon reasonable request. The data are not publicly available due to privacy or ethical restrictions.

## Footnotes

1 The absence of zero in the confidence intervals suggests a significant indirect effect.
